# The Effects of Data Collection Method and Monitoring of Workers’ Behavior on the Generation of Demolition Waste

**DOI:** 10.3390/ijerph14101216

**Published:** 2017-10-12

**Authors:** Gi-Wook Cha, Young-Chan Kim, Hyeun Jun Moon, Won-Hwa Hong

**Affiliations:** 1 Department of Architectural Engineering, Dankook University, 152 Jukjeon-ro, Suji-gu, Yongin 16890, Korea; cgwgnr@gmail.com (G.-W.C.); hmoon@dankook.ac.kr (H.J.M.); 2 Innovative Durable Building and Infrastructure Research Center, Hanyang University, 55 Hanyangdaehak-ro, Sangnok-gu, Ansan 426-791, Korea; 3 School of Architecture, Civil, Environmental and Energy Engineering, Kyungpook National University, 80 Daehak-ro, Buk-gu, Daegu 41566, Korea; hongwonhwa@gmail.com

**Keywords:** construction and demolition waste (C&DW), demolition waste generation rate (DWGR), demolition waste management strategy, monitoring of behavior, data collection method

## Abstract

The roles of both the data collection method (including proper classification) and the behavior of workers on the generation of demolition waste (DW) are important. By analyzing the effect of the data collection method used to estimate DW, and by investigating how workers’ behavior can affect the total amount of DW generated during an actual demolition process, it was possible to identify strategies that could improve the prediction of DW. Therefore, this study surveyed demolition waste generation rates (DWGRs) for different types of building by conducting on-site surveys immediately before demolition in order to collect adequate and reliable data. In addition, the effects of DW management strategies and of monitoring the behavior of workers on the actual generation of DW were analyzed. The results showed that when monitoring was implemented, the estimates of DW obtained from the DWGRs that were surveyed immediately before demolition and the actual quantities of DW reported by the demolition contractors had an error rate of 0.63% when the results were compared. Therefore, this study has shown that the proper data collection method (i.e., data were collected immediately before demolition) applied in this paper and monitoring on the demolition site have a significant impact on waste generation.

## 1. Introduction

Waste generated during construction and demolition (C&D) activities can have considerable environmental implications [1] because these consist of materials including concrete, bricks, excavated soil metals, glass, wood, plastic asbestos, and others [2]. The accurate estimation of C&D waste is very important because it enables the government (or municipality) and the contractor to plan waste control strategies [3]. Consequently, the importance of the optimal management of C&D waste (C&DW) has been considered in many studies [4]. The waste generation rate (WGR) is used widely for predicting the amount of generated C&DW and for optimizing C&DW management (C&DWM) [5]. Hence, the WGR in the field of C&DWM has been considered a fascinating subject of study by many researchers [6,7,8,9,10,11,12,13]. Recently, Lu et al. (2016) developed an S-curve model for WGR prediction by applying the artificial neural networks (ANN) method, and the mean square error of the prediction model showed a low prediction error of less than 5%. In general, an empirical quantity of C&DW can be predicted using the WGR, which can facilitate optimal C&DWM (e.g., environmental impact assessment, prediction of waste disposal charges, recycling practices, and estimation of pick-up truck requirements) [14].

In order to obtain the generation rate of C&DW, data must be collected beforehand. Data collection methods can be categorized into two main types [10,15]: (a) indirect measurements (methods that use data based on the amount of material used during construction and on existing research papers, and (b) direct measurements (methods that measure C&DW on site). However, the former cannot reflect changes in the quantity of material resulting from building renovation, and the latter can introduce errors attributable to the distortion of C&DW information by C&DW contractors [16]. One of the reasons for this distortion is that contractors are not obligated and they do not have the responsibility to accurately report the data (e.g., amount and type) of C&DW generated [17,18]. Consequently, significant discrepancies between C&DW data obtained from the WGR and actual on-site C&DW data can exist. To overcome such a fundamental limitation, Kleemann et al. (2016) proposed a method based on taking direct measurements immediately before building removal and calculating the amount of material [19]. Although this method has some difficulty in acquiring sufficient numbers of samples because of the considerable time and labor required [5,20], it does have the advantage of collecting highly reliable data [19].

Much previous research has focused on the internal factors of buildings such as age, type, purpose of use, and structural and external factors, such as existing work practices, processes and technologies, and duration of demolition as characteristics that affect waste generation [16]. Generally, the literature preceding Teo and Loosemore (2001) considered external factors as having the greatest effect on waste generation [8,21,22,23,24]. Despite external factors being very useful in the consideration of C&DWM, the significance of the effects of the behavior and attitude of workers on waste generation has largely been overlooked [21]. Some researchers recognized this and they introduced a C&DWM strategy based on site management and the monitoring of workers’ behavior [21,25,26,27,28,29]. These studies have proven the importance of monitoring the attitudes of workers involved in C&DWM and the effect it has on waste generation. However, these earlier studies focused mainly on construction waste (CW) and there are few studies based on demolition waste (DW). Furthermore, the scope of the literature is limited because it primarily relies on interviews and the results of questionnaire surveys with on-site workers.

For optimal C&DWM, adequate and reliable WGR data must be obtained and the accuracy of the WGR must be verified through on-site investigation. In addition, the effects of site management and of monitoring the behavior of workers on waste generation must be verified empirically. Hence, in this study, data were collected via direct measurement of DW from C&DW immediately before building removal. The DW generation rate (DWGR) was derived from 423 residential buildings within the area of study: 342 buildings from area A and 81 buildings from area B. Furthermore, the entire demolition process for area B was monitored as a part of the site management to empirically verify its effect on DW generation. Here, the project-level (area A and B) estimations obtained from the derived DWGRs are compared with data reported by a demolition contractor. Based on this, the accuracy of the data collection method used in this study is verified and the effect of monitoring on DW generation is discussed. Lastly, discussions on the results and recommendations regarding measures for predicting waste generation are presented.

## 2. Literature Review

As briefly mentioned in Section 1, an appropriate data collection method is crucial for obtaining reliable WGR data. Furthermore, waste generation is affected by site management, including the attitude of workers during the demolition process [1]. Therefore, this Section investigates previous studies related both to data collection methods for deriving WGRs and to an existing C&DWM method that uses site management, including workers’ attitudes during the demolition stage.

### 2.1. Waste Management Strategies Involving Monitoring and Site Management

In many previous studies, it has been considered that C&D activity is affected by the workers [21,26,27,28,29]. Much research related to C&DWM using monitoring and site management has been based on interviews and questionnaire surveys conducted on construction sites. Lingard et al. (2000) undertook a survey of employees’ perceptions of contracting firms’ waste management systems in the construction industry [25]. Their results showed that management’s interest in waste management and the actions taken to implement company policy should be highly publicized to ensure that the on-site construction workers fully comprehend the management’s commitment. Furthermore, the findings also provided some valuable insights on waste for the construction industry. Teo and Loosemore (2001) used a survey to investigate the attitudes toward waste, and the main influences upon those attitudes, of workers in the construction industry [21]. Based on their findings, they suggested measures for improving workers’ attitudes toward waste in the construction industry. Kulatunga et al. (2006) investigated the attitudes and perceptions of a construction workforce regarding construction waste in Sri Lanka using a questionnaire survey [26]. The results of this study highlighted the perceptions and attitudes of the construction workforce toward waste management as a waste-reducing measure. On the other hand, the study identified that a negative attitude and a lack of awareness toward reducing waste in the construction industry hinders appropriate waste management. Based on interviews conducted in India, Arif et al. (2012) stated that client preference and the enforcement of existing laws facilitated waste minimization [28]. Furthermore, they mentioned that the lack of awareness and education are major factors that hinder waste minimization on construction sites. Having conducted a questionnaire survey in Vietnam, Yean Yng Ling el al. (2013) emphasized the importance of the supervision of contractors and workers on construction sites in waste minimization [29]. Furthermore, based on questionnaires and a literature review, Esa et al. (2017) introduced various strategies for minimizing CW and DW [28]. They showed that site management and the monitoring of the attitudes of workers are crucial for restricting waste generation during construction and demolition stages.

These previous studies have been highly valuable in suggesting management strategies for reducing waste generation during construction. Such management strategies can also be applied to the demolition process. Despite the increasing mechanization of demolition processes, they remain highly labor intensive. Furthermore, the scale of waste generated during demolition is usually larger than during construction. Hence, the objective of this study was to achieve empirical verification through the comparison of data from sites both with and without strategies during building demolition. Thus, this study analyzed the effects of monitoring and of site management on the amount of waste generated on demolition sites.

### 2.2. Methods for Collecting WGR Data

Many studies have applied the WGR method to predict the amount of waste generated during the construction, renovation, and demolition processes. This method can be very useful for predicting and understanding waste generation at both the project and region levels [16,30]. Typically, the WGR can be explained from two perspectives: (1) classifying waste into different categories, and (2) treating the WGR as a whole [5]. Researchers such as Poon et al. (2004a), Lau et al. (2008), Forsythe and Marsden (1999), Formoso et al. (2002), Treloar et al. (2003), and Tam et al. (2007) have all used method (1) to derive WGRs according to different waste types [6,8,12,22,31,32]. This method derives the WGR for each specific material as a percentage of the total amount of waste generated. The WGR obtained using method (1) allows specific values for different waste types to be studied according to building characteristics, material handling process, and waste treatment [5]. Conversely, other researchers [11,33] have chosen not to separate the different materials but have instead used volume (m^3^) or mass (ton) for the total amount of waste generated for each gross floor area (GFA) per m^2^ to obtain the WGR. The WGR obtained from such a method is useful for understanding the total amount of waste generated from single or multiple projects [5,16].

The WGR can be expressed in many different units of measurement depending on the properties of the different materials [5,21]: (1) percentage of purchased material or material needed for a design, (2) kg/m^2^ of GFA, and (3) m^3^/m^2^ of GFA. For example, the GFA can be multiplied by the WGR (kg/m^2^ or m^3^/m^2^) of (2) to calculate the total amount of generated waste. Alternatively, the WGR (percentage of each material in terms of the total amount of generated waste) from (1) can be analyzed to calculate the amount of waste generated for each material. However, when predicting waste generation at a regional level using such a method, the various characteristics and types of buildings that exist within that region cannot be reflected. Therefore, as in the study by Cochran et al. (2007), the differences in construction techniques must be reflected (e.g., various wall materials and roof materials found in buildings with the same structure) when predicting the amount of waste generated at a project level [18].

Various types of data collection method have been implemented in studies of WGRs: (a) indirect measurements (methods using existing data) and (b) direct measurements (methods measuring C&DW on site). Using method (a), Zhao et al. (2011) investigated WGRs with data obtained from the Chongqing Yearbook (a large annual statistical publication compiled by the Chongqing Municipal Bureau of Statistics) [34]. Coelho and de Brito (2012) conducted a study related to C&D flow distribution based on data from existing studies [15]. Hsiao et al. (2002) used existing statistical data to develop a dynamic model of domestic material flows of concrete waste in order to calculate C&DW generation [35]. However, method (a) is unable to properly reflect the changes in the quantity of material resulting from renovation work that might occur during the lifespan of a building. In addition, according to Lu et al. (2011), method (b) can be broken down further into two methods: (b-1) hard methods such as sorting and weighing on site and truck load records, and (b-2) soft methods such as questionnaire surveys and interviews. For example, in collecting their data, Poon et al. (2004a) used tape measurements and truck load records, Poon et al. (2001) and Formoso et al. (2002) used direct observations, and Lau et al. (2008) and Wu et al. (2014) used direct measurements, which all fall under method (b-1). Conversely, in collecting their data, Wang et al. (2010) used questionnaire surveys and Treloar et al. (2003) and Tam et al. (2007) used interviews, which all fall under method (b-2). However, as mentioned in Section 1, data on the quantity of generated waste can be distorted by demolition contractors when using method (b) [16,17]. Consequently, Kleemann et al. (2016) proposed a method to investigate the material composition of a building immediately before its demolition [19]. This method has the advantage of collecting data that are more objective but it is time consuming; thus, its limitation is in acquiring only a relatively low number of samples with which to derive the WGR [16].

Based on the results of a literature review, this study derived the WGR by verifying the reliability of data using the following methods. (1) The measurement method for the WGR used appropriate units, such as kg/m^2^, m^3^/m^2^, and m^2^/m^2^ of GFA according to the characteristics of the material; (2) Data were collected immediately before demolition by conducting a complete enumeration survey on the quantity of material; (3) Sufficient data were collected to overcome the difficulties associated with insufficient sampling; (4) The derived WGR was used according to building type (e.g., building structure, wall material, and roof material) and material (different categories) at the project level to predict the amount of waste generated at the regional level.

## 3. Methodology

This Section discusses the target on-site area, data collection method, and monitoring strategy including site management and workers’ behavior required for the current study.

### 3.1. Target Area and Data Collecting Method

This study focused on the cities of Daegu and Busan in Korea to study the differences in waste generation arising from the effects of the data collecting method and monitoring adopted during the demolition process when deriving the WGR. The target area was divided into two: area A (number of buildings: 324, total GFA: 33,157 m^2^, Daegu) and area B (number of buildings: 81, total GFA: 13,327 m^2^, Busan). The target area was selected for the following reasons. (1) Old buildings were concentrated in this target area and the Korean government was implementing a policy of building renewal at the time of the study. Therefore, the target area was appropriate for calculating the amount of material by taking direct measurements immediately before the building removal process began; (2) Most of the old buildings within the target area comprised low-rise constructions. The amount of waste generated from the demolition of old low-rise buildings in cities within Korea is expected to increase in the future. Thus, consideration of such activities has immediate relevance. Furthermore, in order to study the difference in waste generation in relation to management and monitoring, managers and researchers supervised the entire demolition process (building demolishment, collection and loading of DW, measurement of DW, and removal of DW) in area B.

**Figure 1:**
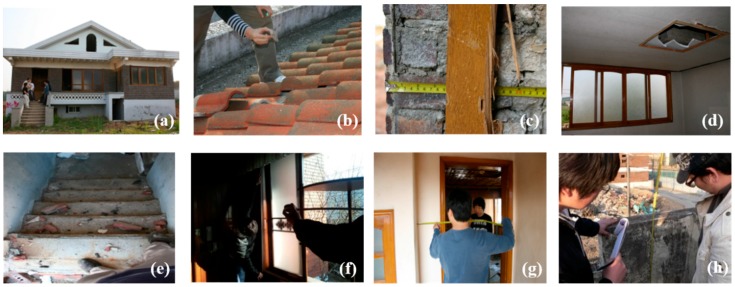
Taking measurements of building characteristics at different locations: (**a**) surveying general characteristics of a building; (**b**) roof; (**c**) wall; (**d**) floor and ceiling; (**e**) stairs; (**f**) windows; (**g**) door; and, (**h**) wall/fence.

**Figure 2:**
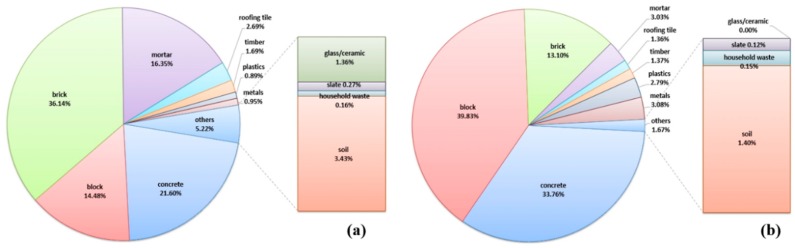
Distribution of demolition waste (DW) generation: (**a**) area A; (**b**) area B.

The method for securing reliable data, as mentioned in Section 2.2, was used in this study to conduct a complete enumeration survey of all of the individual buildings within the target area to obtain the WGR, as shown in Figure 1. The building survey was performed by pairs of people who took direct measurements and completed a data sheet listing the buildings’ characteristics, major components (e.g., roofs, walls, floors, ceilings, stairs, windows, doors, and fences), and materials. The quantities of the main materials were surveyed by measuring lengths, heights, thicknesses, and shapes. Here, the data obtained by the direct measurements were recorded on the data sheet together with floor plans using AutoCAD (Autodesk, San Rafael, CA, USA), and then they were added up to calculate the overall quantities of the main components. The recording sheet used for compiling the data comprised elements for noting the floor plans and general characteristics of the buildings (region, address, use, structure, wall material, roof material, GFA, and the number of floors), recording quantities of each main material and their summed values, and specifying 12 possible types of DW (concrete, brick, block, mortar, slate, timber, roofing tile, glass and ceramics, plastics, household waste, metals, and soil). Figure 2 shows the distribution of DW generation within areas A and B. Table 1 shows the details of the buildings located within areas A and B obtained from the complete enumeration surveys.

### 3.2. Calculating DWGRs for Different Building Types and Method Used for Estimating Regional-Level DW Generation

The DWGR in this study was determined from the DWGR of each property surveyed and the average GFA of the corresponding building. In addition, as in Wang et al. (2004) and Cochran et al. (2007), it was assumed that 100% of the material of the building was produced as waste from the demolition activities [18,36]. However, when the scale of demolition is large, various types of building exist within the same region. Therefore, as mentioned before, a DWGR that can reflect the various construction techniques (e.g., structure, structure material, roof material) used in a building is required in order to predict the amount of project-level waste generated. Consequently, this study collected data by considering the different construction techniques applied to the buildings, as shown in Table 1. Furthermore, based on the collected data, the DWGR was calculated using Equation (1), in which the type of building and the properties of the DW were considered:

(1) $$DWGR_{i}\;of\;k\;type\;building\;=\;\frac{\sum A_{ij}\;of\;k\;type\;building}{GFA\;of\;k\;type\;building},$$

where $A_{ij}$ is the amount of material *j* with properties of waste material *i* (quantity) (m^3^, or m^2^, or ton), and GFA is the gross floor area (m^2^).

The DW generated from the target area (area A and area B) was categorized according to the building types located within the same area. Then, the DWGRs for the different waste categories (13 types of waste) were derived using the GFA. The DW was calculated using Equation (2), as shown below:

(2) $$\begin{array}{l} \mathrm{DW}\;\mathrm{estimation}\;\mathrm{of}\;\mathrm{project-level}\;\\ \;=\overset{n}{\underset{k=1}{\sum }}\overset{12}{\underset{i=1}{\sum }}(DWGR_{i}of\;k\;type\;building\times GFA\;of\;k\;type\;building)\;+\;\mathrm{Mo} \end{array}$$

where DWGR*_i_* is the WGR (m^3^/m^2^, or m^2^/m^2^, or ton/m^2^) of waste with property *i* and Mo is the amount of waste generated from buildings that did not have sorting standards.

This study distinguished the measuring units of waste based on weight (metals), volume (concrete, brick, block, mortar, timber, glass/ceramic, soil, and household waste), and surface area (roofing tile and slate) to survey the DWGRs. However, the DW data reported by the demolition contractor was provided in units of weight. Consequently, the units of the surveyed DWGRs were converted into units of weight. This study used density values as conversion factors for each material, as shown in Table 2, to calculate the weight of the DW.

### 3.3. Strategies for Monitoring the Site and Workers’ Behavior

**Figure 3:**
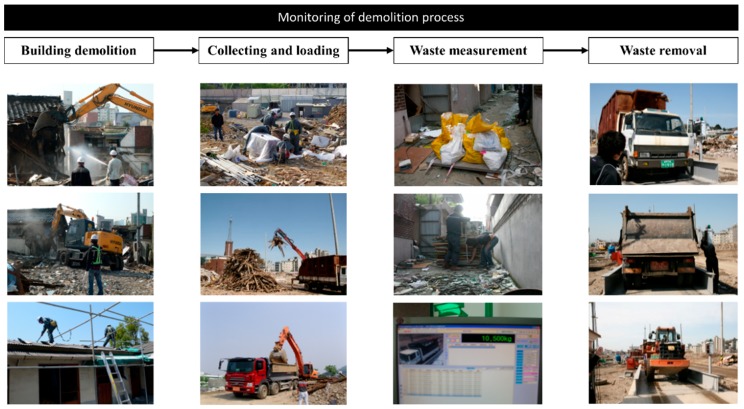
Supervising strategy adopted for area B included recording videos of the demolition site and monitoring of the site by managers and researchers.

As mentioned before, this study attempted to investigate the effect of monitoring the workers and their behavior during demolition on DW generation. For this, the contractor responsible for the demolition of area A was unrestricted in undertaking the demolition process. However, for area B, managers and researchers supervised the site throughout the entire demolition process. The supervising strategy adopted for area B included recording videos of the demolition site and monitoring of the site by managers and researchers (see Figure 3). In addition, the workers on site were notified that the construction site was supervised. Mechanized demolition methods were used for areas A and B, and the same type of equipment was used for collecting and loading DW.

## 4. Results and Discussion

### 4.1. Generation Rates and Composition of DW

Table 3 shows the results of the DWGR survey according to the different types of building within the target area. The overall mean of area A was 1209 kg/m^2^. However, the buildings in area A were categorized into 12 types; thus, the DWGR results also varied. The DWGR from building type Re/Co-RC-BR-S within area A was the highest at 1842.9 kg/m^2^ and the DWGR from wooden buildings was the lowest: 739.4 kg/m^2^ for type Re-W-BR-RT and 746.8 kg/m^2^ for type Re-W-BL-RT. The overall mean of area B was 1402.3 kg/m^2^. Area B had few BLS buildings, which have low DWGR results, and it had no wooden buildings; thus, the overall mean was higher when compared with area A. The buildings in area B were categorized into four types: the DWGR from building type Re/Co-RC-BL-S+RT was highest at 1618.0 kg/m^2^ and the DWGR from building type Re-BLS-BL+RT was lowest at 1005.4 kg/m^2^.

**Figure 4:**
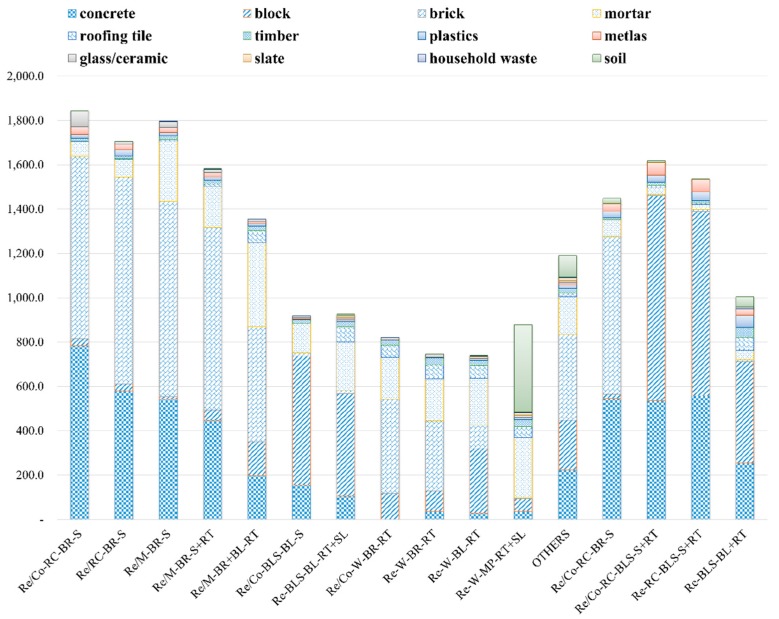
The DWGRs of different building types.

**Figure 5:**
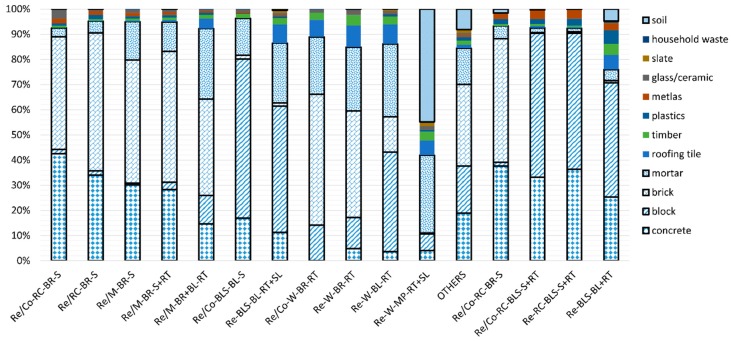
Distribution of DW generation for different building type.

The results of surveying the DWGR for the different building types in areas A and B showed that DWGR according to the actual use of the buildings differed, even if the buildings had the same structure, wall material, and roof material. For example, the DWGR according to building type for area A (Re/Co-RC-BR-S: 1842.9kg/m^2^, Re/RC-BR-S: 1706.5 kg/m^2^, Re/Co-W-BR-RT: 822.0 kg/m^2^, and Re-W-BR-RT: 746.8 kg/m^2^) and area B (Re/Co-RC-BLS-S+RT: 1618.0 kg/m^2^ and Re-RC-BLS-S+RT: 1537.4 kg/m^2^) was slightly higher for buildings that had both residential and commercial purposes, as shown in Figure 4. However, even though the DWGR according to building types differed, the compositions were very similar. Furthermore, the DWGR from areas A and B differed, even if the building type (e.g., Re-RC-BR-S) was identical. Therefore, it is anticipated that DWGR will be different according to regional characteristics, even if building characteristics are identical. However, the composition of building type Re-RC-BR-S turned out similar, as shown in Figure 5.

Among the various compositions, brick generated the highest DWGR with an overall mean of 437 kg/m^2^ in area A. This is because brick was used as the wall material in many building types in area A. The next highest DWGR was concrete with an overall mean of 261.1 kg/m^2^; this material was used for structural frameworks of buildings, foundations, and slabs. Materials that constitute the main structures, floors, and walls (concrete, block, brick, mortar, and soil, which is a typical wall material in traditional Korean wooden buildings) comprised a large proportion of the total DWGR. For area B, block constituted the largest DWGR with an overall mean of 558.6 kg/m^2^. This was followed by concrete and brick with overall means of 473.46 and 183.76 kg/m^2^, respectively. As in area A, the materials used for the main structural elements, floors, and walls of the buildings in area B comprised the largest proportion of the total DWGR.

### 4.2. Comparison of Compositional Data with Demolition Contractors’ Reports

In this section, the DWGRs derived in this study were applied to compare the estimated quantities of DW for areas A and B with those reported by the demolition contractors. Only four types of DW were reported by the demolition contractors, whereas 12 types were surveyed during this study. The demolition contractors categorized DW as mineral waste (including concrete, block, brick, mortar, and roofing tile), timber, plastics, and mixed waste. Mineral waste, which comprises the largest proportion of waste from a demolition process, is important for predicting significant amounts of DW produced at district or regional levels. The results are shown in Table 4.

The results of applying the surveyed DWGRs showed that area A (without monitoring during the demolition process) reported a total that was 7582 tons (18.5%) more than the estimated DW. However, area B (monitored throughout the demolition process) reported a total that was 747 tons (4.0%) less than the estimated DW. The amount of mineral waste generated was 19.6% (9197 tons) more than estimated for area A and approximately 3.0% (499 tons) less than estimated for area B. Overall, the estimated DW, based on the surveyed DWGRs, and the reported data in area B were similar. In addition, for the generated amount of wood waste, area A showed a significant error rate (ER) of 71% between the estimated DW and the reported data, whereas area B showed similar results between the two values with an ER of 2.2%. The results showed that monitoring throughout the demolition process had a demonstrable effect on the amount of waste generated. However, in this study, despite the same data collection method being applied, there are some significant discrepancies and variations between the estimated and reported values for DW categories and mass. The reason for this seems to be probably the combination of various external factors (e.g., labours’ attitude and skill, demolition contractors’ unfaithful disclosure, C&D time, economic situation of demolition contractors, etc.).

The amounts of generated DW in other categories showed large discrepancies. As shown in Table 4, plastic showed significant differences between the estimated DW and reported data for both area A and area B. In addition, some DW was not reported by the unsupervised demolition contractor for the following reasons. First, the demolition contractor was not obligated to provide an accurate report on all DW types, as mentioned in previous studies by Fatta et al. (2003), Kleemann et al. (2016), and Chen et al. (2017). Second, as in the study by Kleemann et al. (2016), there could have been inconsistencies between the waste categories used by the demolition contractor and the theoretical waste categories or categories set by the government. For example, in Korea, the category of waste currently used by demolition contractors is relatively simple and it generally comprises mineral waste (including concrete, mortar, brick, block, and roofing tile), flammable waste, inflammable waste, and mixed waste. Third, waste with a high recycle value (plastics or wood without impurities, and metals) was sold to recycling companies during the demolition; hence, it was not reported properly post-demolition. It was the same case for this study, where metal waste specifically was not reported by the unsupervised demolition contractor, who confirmed the sale of all metals to a recycling company. Lastly, as shown in Table 4, the amount of mixed waste generated was high. Large-scale demolition is typically performed using mechanized methods; thus, building waste that is produced in small volumes or that is difficult to sort (e.g., plastics, glass, ceramics, slate fragments, and soil) is usually produced in mixed form. 

Kleemann et al. (2016) highlighted the challenges associated with obtaining reliable data from contractors. However, a very accurate prediction was made in this study for area B regarding the quantity of generated mineral waste, which constitutes the largest proportion of DW, by applying the data collection method and monitoring. In addition, the estimated and actual quantities of generated wood waste had a significantly low ER of 2.2%. However, the results for area A showed high ERs in most waste categories (see Table 4). Moreover, the quantities of waste generated from area B had an ER of 0.63% when the results were compared with the exclusion of metals and slate. In other words, the estimates obtained by applying the DWGRs surveyed in this study and the reported quantities of generated waste showed no discrepancies (Table 5).

## 5. Recommendations

As shown in Table 3, old buildings within cities are generally comprised of various types. This is because the buildings were built during different economic times and many of them were extended or remodeled. For example, the original wall material (e.g., wood or mud) of many of the traditional Korean wooden buildings within the target area had been replaced with bricks or blocks. Furthermore, the roofs of traditional wooden buildings are usually composed of roof tiles, but there were cases where the roof tiles had been replaced by slate during remodeling. Moreover, many buildings had walls and roofs composed of many different materials rather than one single material. Therefore, such factors must be considered in order to predict DW generation accurately.

Mineral waste (concrete, brick, block, mortar, and cement roofing tile) constitutes the largest proportion of DW; hence, it is the most important type of waste regarding the prediction of DW generation at district and regional levels. If the results of area B were considered in isolation, it would be possible to categorize mineral waste adequately using mechanical demolishing methods with appropriate monitoring. Furthermore, the fact that concrete, brick, block, mortar, and cement roofing tile waste are usually categorized simply as mineral waste by demolition contractors requires attention. As metals, wood, and plastic have high recycle values (as long as the latter two materials have no impurities), demolition contractors have the tendency to sell these materials before or during the demolition process. Therefore, to account for the quantity of this waste properly, it would be necessary to track these materials to the recycling company that had a contract with the demolition contractor. Furthermore, continuous monitoring throughout the entire demolition process should be performed on the route taken by the pick-up trucks during waste removal.

Waste produced in small volumes or waste that is difficult to sort is mostly cleared as mixed waste during building demolition. The monitoring of area B showed that mixed waste accounted for up to 6.63% of the total waste generated. This seemed to be attributable to the demolition method used by the contractor and to the lack of awareness of the workers regarding the importance of sorting waste before its removal. Hence, in order to reduce the quantity of mixed waste, monitoring of the entire demolition process and striving to raise the awareness of the workers regarding waste will be required.

## 6. Conclusions

This study surveyed DWGRs for different types of building by conducting on-site surveys immediately before demolition in order to collect adequate and reliable data. In addition, the effects of DW management strategies and of monitoring the behavior of workers on the actual generation of DW were analyzed. The results showed that when monitoring was implemented, the estimates of DW obtained from the DWGRs that were surveyed immediately before demolition and the actual quantities of DW reported by the demolition contractors were similar. Therefore, it can be concluded that the survey method adopted to derive the DWGRs and the monitoring of the site throughout the demolition process are strategies that are extremely useful for managing and reducing waste. However, as mentioned above, there were some discrepancies between the reported by demolition contractors and predicted values. This can be attributed to various uncertain factors in the field. Thus, uncertainty is a major challenge and it needs to be considered for accurately estimating waste generation.

## Figures and Tables

**Table 1:** States of different types of building located within the surveyed target areas.

Location	Usage	Structure	Wall Material	Count	GFA (m^2^)	Building Types
area A	Residential/Commercial	Reinforced concrete	Brick	16	3519	Re/Co-RC-BR-S
	Residential	Reinforced concrete	Brick	12	1895	Re/RC-BR-S
	Residential/Commercial	Masonry	Brick	8	931	Re/M-BR-S
	Residential	Masonry	Brick	73	7950	Re/M-BR-S+RT
	Residential	Masonry	Brick +block	4	355	Re/M-BR+BL-RT
	Residential/Commercial	Block	block	3	296	Re/Co-BLS-BL-S
	Residential	Block	block	127	9311	Re-BLS-BL-RT+SL
	Residential/Commercial	Wood	Brick	2	135	Re/Co-W-BR-RT
	Residential	Wood	Brick	19	1948	Re-W-BR-RT
	Residential	Wood	block	33	3324	Re-W-BL-RT+S
	Residential	Wood	MP	27	2104	Re-W-MP-RT+SL
	Others			18	1408	OTHERS
area B	Residential/Commercial	Reinforced concrete	Brick	8	1394	Re/Co-RC-BR-S
	Residential/Commercial	Reinforced concrete	block	7	1823	Re/Co-RC-BL-S+RT
	Residential	Reinforced concrete	block	25	6501	Re-RC-BL-S+RT
	Residential	Block	block	41	3609	Re-BLS-BL+RT

Re: residential; Co: commercial; RC: reinforced concrete; M: masonry; BLS: block frame; W: wood frame; BR: brick; BL: block; MP: mud-plastered; S: slab; SL: slate; RT: roofing tile; GFA: gross floor area.

**Table 2:** Material density values used in this study.

Material Type	Density Values	Unit	Description
Concrete	2300	kg/m^3^	construction standard production unit system
Block	1900	kg/m^3^	construction standard production unit system
Brick	2000	kg/m^3^	construction standard production unit system
Mortar	2000	kg/m^3^	construction standard production unit system
Timber	590	kg/m^3^	construction standard production unit system for weight of dry wood
Slate	10.5	kg/m^2^	10.5 kg/m^2^ was used for weight per unit surface area of cement asbestos containing 10% asbestos
Roofing tile	198	kg/m^2^	one cement roofing tile: 5.5 kg, calculated for 36 tiles/m^2^
Glass/ceramic	2300	kg/m^3^	densities of sheet glass and ceramics range between 2100 and 2500 kg/m^3^, so an average value of 2300 kg/m^3^ was used
Plastics	1200	kg/m^3^	density of plastic used in buildings ranges between 900 and 1500 kg/m^3^, so an average value of 1200 kg/m^3^ was used
Household waste	300	kg/m^3^	density value of 300 kg/m^2^ was used for household waste according to a performance guarantee process guideline for processing abandoned waste, Regulation No. 249 by the Ministry of Environment
Soil	1600	kg/m^3^	dry soil weight

**Table 3:** The demolition waste generation rates (DWGRs) of different building type (kg/m^2^).

Building Types		Waste Types												
		Concrete	Block	Brick	Mortar	Roofing Tile	Timber	Plastics	Metals	Glass/Ceramic	Slate	Household Waste	Soil	Total
Project A	Re/Co-RC-BR-S	783.8	31.0	825.3	64.8	2.1	12.7	17.7	34.7	69.9	-	0.9	0.0	1842.9
	Re/RC-BR-S	580.5	29.9	933.3	80.4	4.4	9.5	28.8	25.2	12.1	0.3	1.6	0.4	1706.5
	Re/M-BR-S	540.9	13.3	880.1	272.4	6.3	17.1	15.6	23.5	24.1	0.2	3.7	-	1797.1
	Re/M-BR-S+RT	447.6	46.0	824.1	185.1	11.6	16.0	15.2	19.6	13.5	1.0	1.9	2.0	1583.6
	Re/M-BR+BL-RT	197.6	153.2	519.3	379.8	53.2	21.1	11.6	8.6	9.3	-	1.6	-	1355.3
	Re/Co-BLS-BL-S	155.4	580.5	15.2	134.0	-	16.7	1.3	6.9	7.8	-	1.2	-	919.0
	Re-BLS-BL-RT+SL	104.8	464.0	11.9	220.2	69.1	23.6	5.8	4.7	8.3	7.7	1.6	4.9	926.6
	Re/Co-W-BR-RT	-	116.2	427.3	187.5	55.6	23.1	0.4	-	10.9	-	1.1	-	822.0
	Re-W-BR-RT	35.9	92.0	316.3	189.7	64.2	30.5	1.8	1.6	13.4	0.0	1.1	0.1	746.8
	Re-W-BL-RT	26.8	292.9	103.1	214.0	57.8	22.7	7.2	1.2	7.9	3.6	2.2	0.1	739.4
	Re-W-MP-RT+SL	36.1	57.2	3.0	272.8	49.9	31.7	7.3	1.6	10.3	11.5	3.8	394.0	879.2
	OTHERS	224.2	224.4	385.0	171.3	15.7	20.6	16.4	9.8	9.2	14.1	2.7	96.6	1190.0
	Overall mean	261.1	175.1	437.0	197.7	32.5	20.4	10.8	11.4	16.4	3.2	1.9	41.5	1209.0
Project B	Re/Co-RC-BR-S	544.4	21.7	711.6	73.7	-	9.5	29.6	32.7	-	2.1	-	23.2	1448.5
	Re/Co-RC-BL-S+RT	537.0	923.5	6.5	29.9	11.2	13.1	32.7	55.8	-	2.4	-	5.9	1618.0
	Re-RC-BL-S+RT	558.1	832.2	8.1	22.1	7.3	9.6	39.8	57.1	-	1.0	-	2.0	1537.4
	Re-BLS-BL-RT	253.9	456.8	8.7	44.4	57.7	44.9	54.6	27.4	-	1.1	8.1	47.6	1005.4
	Overall mean	473.4	558.6	183.7	42.5	19.0	19.3	39.2	43.2	-	1.7	2.0	19.7	1402.3

**Table 4:** Comparison of estimated DW with that reported by demolition contractors (unit: ton).

Waste Type		Project A			Project B (Monitored)		
		Estimated in This Study		Reported	Reported	Estimated in This Study	
mineral waste	concrete	9563	37,645	46,842	16,381	16,880	6282
	block	6695					8773
	brick	13,908					1088
	mortar	6251					461
	roofing tile	1228					276
timber		671		195	256	262	
plastics		377		198	24	557	
glass/ceramic		570		-	-	no data	
metals		421		Not reported	Not reported	617	
Soil		1029		-	-	228	
Slate		137		106.2	Not reported	18	
household waste		62		-	-	29	
mixed waste		-		1151	1183	-	
total		40,911		48,492	17,844	18,591	

**Table 5:** The comparison of waste generation between estimated and reported total DW Generation in project A and B.

Target Area	Including All Wastes			Excluding Wastes Which Were Not Reported		
	Estimated Total DW Generation	Reported Total DW Generation	Error Rate (%)	Estimated Total DW Generation	Reported Total DW Generation	Error Rate (%)
**Project A**	40,911	48,492	18.5	40,353	48,492	16.8%
**Project B**	18,591	17,844	4.0	17,956	17,844	0.63%
